# The psychological well-being of Norwegian adolescents exposed in utero to radiation from the Chernobyl accident

**DOI:** 10.1186/1753-2000-5-12

**Published:** 2011-04-17

**Authors:** Kristin Sverdvik Heiervang, Sarnoff Mednick, Kjetil Sundet, Bjørn Rishovd Rund

**Affiliations:** 1 Department of Psychology, University of Oslo, P.O.Box 1094 Blindern, NO-0317 Oslo, Norway; 2 Akershus University Hospital, Department of Research & Development, Division Mental Health, Norway; 3 Psychology Department, University of Southern California, Los Angeles, California 90089-0375, USA; 4 Vestre Viken Hospital Trust, Norway

## Abstract

**Background:**

On 26 April 1986, the Chernobyl nuclear power plant suffered an accident. Several areas of central Norway were heavily affected by far field radioactive fallout. The present study focuses on the psychological well-being of adolescents who were exposed to this radiation as fetuses.

**Methods:**

The adolescents (n = 53) and their mothers reported their perceptions of the adolescents' current psychological health as measured by the Youth Self Report and Child Behaviour Checklist.

**Results:**

In spite of previous reports of subtle cognitive deficits in these exposed adolescents, there were few self-reported problems and fewer problems reported by the mothers. This contrasts with findings of studies of children from the former Soviet Union exposed in utero, in which objective measures are inconsistent, and self-reports, especially by mothers, express concern for adolescents' cognitive functioning and psychological well-being.

**Conclusion:**

In the current paper, we explore possible explanations for this discrepancy and suggest that protective factors in Norway, in addition to perceived physical and psychological distance from the disaster, made the mothers less vulnerable to Chernobyl-related anxiety, thus preventing a negative effect on the psychological health of both mother and child.

## Introduction

The accident at the nuclear power plant in Chernobyl on 26 April 1986 released large amounts of radioactive materials. Several areas of central Norway were heavily affected by far field radioactive fallout. The present study focuses on the individuals who were exposed to the radiation in these areas as fetuses. It is well documented that in utero exposure to a range of environmental toxins may have long-term consequences for neurodevelopment. Most studies have looked into the neurodevelopment effects of exposure to drugs, alcohol and cigarettes. In utero ionizing radiation exposure has received much less attention [1]. The effect of low-dose radiation on the fetus is unclear, and previous research on the neurological and psychological effects of in utero exposure to Chernobyl radiation has been inconsistent.

While the focus has been on the possible cognitive outcomes of in utero exposure to ionizing radiation, there has also been concern about psychological effects. Previous research on children exposed in utero to Chernobyl radiation found a higher incidence of both cognitive and psychiatric problems [2-4]. Other studies of children exposed as infants or in utero did not document any differences between those exposed and controls. However, mothers of in utero exposed children rated their children significantly higher on scales of memory problems, hyperactivity and somatic complaints [5-7]. In Kiev, the overall problem scores on the Child Behavior Checklist were generally high both for children evacuated to Kiev shortly after the accident and for controls who had resided in Kiev before the accident [5,7].

Studies from Hiroshima and Nagasaki indicate that generalized and health-focused anxiety, somatization and depressive symptoms remained elevated for 10 to 20 years after the bombings [5]. Children studied after other disasters, particularly unexpected, severe, traumatic events, have demonstrated increased risk for internalizing and externalizing symptoms [5].

There is evidence for a significant effect of clinical morbidity in certain risk groups after toxicological accidents, especially anxiety disorders in mothers with young children and in evacuees [8]. Women, especially those who have young children to care for, appear to be more at risk for psychological health effects [8]. This heightened vulnerability also affects pregnant women. After the nuclear accident at Three Mile Island, women who lived near the facility and who were pregnant or had young children at the time were among those who experienced the greatest psychological distress [9].

Research of the developmental impact of disasters that involve in utero radiation exposure focus on two main routes of effect--in utero radiological exposures, and the effects of maternal stress on the developing fetus, or a combination of the two [10]. Being exposed to radiation as a result of a power plant accident is a stressful experience for pregnant women. The fact that radiation exposure events usually involve both elevated radiation exposure and higher levels of maternal stress makes it difficult to separate these two routes of effect.

A previous study examining neurocognitive functioning in Norwegian adolescents who were exposed in utero suggested lower IQ [11] and deficits in neuropsychological function compared with nonexposed adolescents [12]. The aim of the current study is to examine the emotional and behavioral functioning of these in utero exposed adolescents, as perceived by the adolescents themselves and their mothers. Will these adolescents and their mothers report elevated levels of problems? Are there significant differences between self-reports and maternal reports?

## Method

### Participants

We recruited 84 adolescents from municipalities in the counties of Oppland and Nord-Trøndelag, which were the areas within Norway most heavily exposed to fallout radiation from the Chernobyl accident. The participants were chosen according to the area of residence of their mothers during pregnancy. All exposed participants were fetuses when the Chernobyl accident took place, or were born within 18 months (0-548 days) after the explosion. The main reason for choosing the 18-month period was the high levels of ionizing radiation in the affected areas during this period; the total exposure reached its maximum about a year after the accident.

Participants were identified through schools in their respective counties. Students in the relevant age range (16.3-20.0 years; median: 18.4 years) were invited to participate through a letter explaining the purpose of the study. All participants were born and educated in Norway and spoke Norwegian as their mother tongue. A questionnaire was distributed to the mothers to determine where they were living during their pregnancies.

Adolescents who met the criteria for a drinking or substance abuse disorder according to the MINI screening module [13] were excluded from this study, as were those who presented evidence of head injuries or significant mental or physical handicaps. Among those who agreed to take part in the study, fifty-three participants returned the YSR and CBCL. These were included in the current study. The other 31 were classified as nonresponders. The demographic characteristics of responders and nonresponders are listed in Table 1. Males and females were equally represented in both groups, the majority of subjects were right handed, and three responders and four nonresponding subjects reported mild psychological problems. Demographic characteristics were not significantly different between the groups.

**Table 1:** Demographic characteristics of the participants

	Responders (*N *= 53)	Nonresponders (*N *= 31)			
	*N/N*	*N/N*	*χ*^*2*^	df, *N*	*P*
Sex (M/F)	27/26	14/17	0.3	1,84	.656
Hand dominance (R/L)	46/7	29*/2*	0.9	1,84	.474
Psychological disorder (Y/N)	3/50	4/26	1.5	1,83	.274
	*M (SD)*	*M (SD)*	*t*	Df	*P*
Age (years)	18.5 (0.6)	18.7 (0.7)	1.3	82	.207
Education (years)	11.4 (0.5)	11.5 (0.5)	0.1	72	.884
Grade level (6 [max]-1 [min]	4.1 (0.7)	3.9 (0.7)	1.6	78	.125
Mother's education (years)	13.3 (2.5)	12.4 (2.4)	1.6	82	.890

External radiation doses were calculated by the Norwegian Radiation Protection Authority (NRPA) from soil deposition patterns. The mean external radiation dose was estimated to equal 0.935 mSv in the exposed group areas during the 18 months following the accident [14]. Because we lack individual measures of exposure of the participants in the current study, individual in utero radiation dosage is considered unknown.

### Measures

Adolescents and their mothers reported mental health problems using the Child Behavior Checklist (CBCL) [15] and its related instrument, the Youth Self Report (YSR) [16]. These are standardized instruments for assessing a broad array of psychopathological manifestations in children, and are among the most widely used for assessing adolescents' emotional and behavioral problems in a variety of settings [17].

The CBCL was designed to tap problems and competencies reported by parents of children aged 5-18, and the YSR measures these problems and competencies as reported by the adolescents themselves, aged 11-18. The CBCL includes 20 competence items, which obtain the parent's report of the amount and quality of their children's participation in sports, hobbies, games, activities, jobs and chores and friendships; how well the child gets along with others; and school functioning. A total score of social functioning can be derived; lower scores indicate poorer functioning. The 118 behavioral items scored on a three-step response scale (0-2) produce a total score that ranges between theoretical limits of 0 and 236. The 2001 version of the scoring program used in the current analyses, generates eight syndrome scale scores: the syndrome scales withdrawn, somatic complaints and anxious/depressed are grouped as "internalizing", and the scales rule-breaking behavior and aggressive behavior are grouped as "externalizing". The internalizing score and the externalizing score are the sum scores of the "internalizing" and the "externalizing" scales, respectively. Numerous studies have provided evidence of the stability of the psychometric properties of the instrument. Moreover, cross-cultural comparisons have yielded relatively small differences in rates of problems and in syndrome structure. The CBCL and YSR have been translated into Norwegian and used extensively in Scandinavia. Previous studies have suggested acceptable reliability and validity for the CBCL for Norwegian adolescents [17,18].

We used the raw scores of the syndrome subscales in the current study. Because Norwegian norms are not available, raw scores are usually reported in Norwegian studies. Using raw scores in the current study made it possible to compare our data with those reported in other Norwegian studies. In order to compute the number of subjects with increased levels of problems and to compare the YSR and CBCL profiles, manual based T-scores were also reported. The assessment took place in 2005 and 2006. Written informed consent was collected from all participants after the procedures were fully explained. The project was approved by the Regional Committee for Research Ethics, and the National Data Inspectorate was notified about the study.

### Statistical analyses

Data were analyzed using SPSS 16.0 for Windows (SPSS Inc., 2007). Group differences in demographic characteristics were subjected to chi-squared analyses (categorical data) and independent sample *t*-tests (continuous data). The Alpha level p < .05 was chosen.

To analyze differences between and within the YSR and CBCL scores, two multivariate repeated measure analyses of variance (MANOVA) were performed in order to control for chance findings due to multiple testing. The first MANOVA was conducted with the responder (YSR or CBCL) and dimension (anxious, withdrawn, somatic complaints, social problems, thought problems, attention problems, rule breaking and aggressive behavior) as the repeating factors. In the second MANOVA, the eight dimension scores were substituted by the three sum scores (Internalization, Externalization and Total Problems score). The seven dimension scores and the three sum scores are medium sized intercorrelated. Hence, F-vaules based on Wilks lambda (Λ) are reported to guard against posible threats to the homogeneity assumption. The two MANOVAs were followed up with paired *t*-test comparisons between the adolescent and mother ratings for each dimension. Level of significanse, p ≤ 0.05, was Bonferroni corrected to guard against type I errors due to multiple testing For profile analysis, raw scores on the Youth Self Report (YSR) by the adolescents and Child Behavior Checklist (CBCL) by the mothers on the eight dimensions and three sum scores were transformed to standardized T-scores (mean: 50, SD: 10) based on the United States standardization sample [16]. The number of individuals who obtained T-scores >60 (i.e., one standard deviation above the mean in the standardization sample) was counted on each dimension and sum score. The number signifies the dimensions and sum scores in which most problems were recognized by the adolescents and their mothers.

## Results

**Figure 1:**
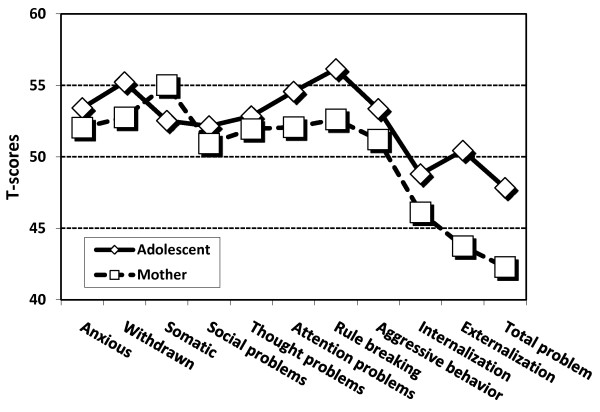
T-scores on Youth Self Report (YSR) by adolescent and Child Behavior Checklist (CBCL) by mother (*N *= 53)

The adolescent self-reports (means and standard deviations) and ratings by their mothers are presented in Table 2. The first MANOVA showed significant main effects on the eight YSR/CBCL dimension scores of both the responder (Λ = 0.40,F (1, 52) = 78.9, p < 0.001), the dimension (Λ = 0.31, F (7, 46) = 14.6, p < 0.001) and the interaction between responder and dimension (Λ = 0.44, F (7, 46) = 8.4, p < 0.001). The second MANOVA also showed significant main effects on the three YSR/CBCL sum scores of the responder (Λ = 0.38, F (1, 53) = 86.7, p < 0.001), the dimension (Λ = 0.34, F (2, 52) = 50.3, p < 0.001), and the interaction between responder and dimension (Λ = 0.37, F (2, 51) = 45.0, p < 0.001). Both MANOVAs indicated that the adolescents reported more problems than their mothers did, but that the differences varied across dimensions, as illustrated by the T-score profiles in Figure 1. Bonferroni corrected paired *t*-tests confirmed significant differences on all dimensions and sum scores, except for Somatic concerns. The adolescents reported most problems on the rule-breaking dimension, followed by the attention dimension, whereas mothers reported most problems on the somatic and anxious dimensions. Among the sum scores, adolescents mostly reported problems on the Externalization score, whereas mothers attributed the problems to the Internalization score.

**Table 2:** Scores on Youth Self Report (YSR) and Child Behavior Checklist (CBCL) (*N *= 53)

Raw scores	YSR *M (SD)*	# T > 60	CBCL *M (SD)*	# T > 60	*t*	df	*P*
Dimensions							
Anxious	3.6 (3.6)	7	1.5 (2.3)	5	5.9	52	<0.001
Withdrawn	3.3 (2.7)	7	1.2 (1.6)	3	7.3	52	<0.001
Somatic	2.1 (1.8)	1	1.5 (2.1)	8	1.8	52	0.08
Social problems	1.8 (2.1)	4	0.4 (1.1)	2	6.0	52	<0.001
Thought problems	2.4 (2.5)	5	0.7 (1.2)	4	5.3	52	<0.001
Attention problems	4.4 (3.7)	8	1.9 (2.5)	2	6.1	52	<0.001
Rule breaking	4.7 (3.8)	15	1.5 (2.2)	4	7.8	52	<0.001
Aggressive behavior	5.4 (4.4)	6	1.8 (2.9)	2	8.5	52	<0.001
Sum scores							
Internalization	9.1 (6.7)	5	4.3 (5.0)	7	6.5	52	<0.001
Externalization	10.2 (7.7)	8	3.3 (4.6)	3	9.2	52	<0.001
Total problems	31.5 (20.9)	4	11.9 (13.4)	3	9.2	52	<0.001

Raw scores, means and standard deviations are reported for each measure. Number of cases (#) obtaining T-scores greater than 60 are listed.

## Discussion

This study assessed in utero exposed adolescents' and their mothers' reports on the adolescents' emotional/behavioral problems. The most noteworthy findings were that in contrast to previous studies of in utero exposed children:

1. The level of problems reported by the adolescents and their mothers was low.

2. The level of problems reported by the mothers was generally lower than that reported by the adolescents.

The current study, with a CBCL mean Total Problems score of 11.8, is in accordance with previous Nordic studies, which also reported low CBCL mean Total Problems scores in comparison with studies in other countries [18]. A Swedish study that examined 1308 school children aged 6-16 years old with the CBCL found a mean Total Problems score of 14.2 [19]. A Norwegian study of 1170 children aged 4-16 [17] found a CBCL mean Total Problems score of 15.4. For the subgroup aged 12-16, the mean Total Problems score was 13.6 [17]. In the current study rates of self-reported behavior problems (YSR) were generally higher for the adolescents, with a mean Total Problems score of 31.1, but within the normal range and lower than those reported in another Norwegian study [20].

The mothers' ratings reported low levels of problems and better psychological functioning than the adolescents reported themselves. Many researchers have reported significant discrepancies between youth-reported and parent-reported psychopathology in adolescents [21]. In studies of nonclinical samples, youths report higher severity ratings than their parents [21]. Our findings are consistent with previous studies comparing parent reports and youth self reports of adolescents' emotional and behavioral problems.

Investigations have documented Chernobyl-related psychological problems in prenatally exposed children in the former Soviet Union [2-4,22]. The causes of these observed psychological problems are uncertain. The radiation release may have a direct, physiological impact on the developing fetal brain, and/or it may affect the fetus in terms of stress on the mother caused by the perceived danger of exposure to Chernobyl radiation. Other stressful consequences of the accident may also continue to affect the child later on. It is difficult to separate the potential impact of these variables.

In the former Soviet Union, the accident had a tremendous impact on the areas surrounding Chernobyl, both in terms of radiation exposure and psychosocial consequences interfering with people's lives. However, some investigations did not document a rise in psychological and behavioral problems in children exposed in utero or as infants in these areas [5,6]. In one study [5], evacuees and non-evacuees obtained high scores on the CBCL problem scale but there were few significant differences between groups. Among the significant differences were maternal ratings of somatic complaints. Evacuee mothers rated their children's well-being as significantly worse, especially on somatic symptoms on CBCL [5]. The most important risk factors for these ratings were somatization and Chernobyl-related stress experienced by the mother. Another study revealed no significant differences between groups related to level of radiation exposure, but mothers who were pregnant at the time of the accident rated their children as significantly more hyperactive [6]. Interestingly, in the Taormina study [7], evacuee mothers were almost three times more likely to report their children as having memory problems.

In the current data mothers rated their children as having fewer problems than the adolescents themselves reported. This pattern is typical in nonclinical groups. This could indicate that the mothers included in the current study were less worried than the mothers in previous investigations of radiation exposed individuals.

People have a strong tendency to worry about their future health once they know they have been exposed to radiation, even when the dose they have received is negligible [23]. The amount of radiation discharged from the accident at Three Mile Island in the United States was less than one-millionth of the release from the Chernobyl accident, but the Three Mile Island accident seriously affected the mental health of the general population [24]. Why does this not seem to apply to prenatally exposed Norwegians and their mothers?

The passage of time may affect the psychological reactions. Five years after the nuclear accident at Three Mile Island, the mental health of women who were living close to the site and were pregnant at the time of the accident was similar to that of women from the same area who became pregnant after the accident. Maternal ratings of the two groups of children when they were five years old were also similar [9]. In a study of in utero exposed children from the former Soviet Union, Korol and Shibata [22] found the prevalence of neurotic disorders to be significantly higher in the in utero exposed group from 1989-1997, but the difference diminished in effect from 1999-2003. These findings suggest that the psychological effects change over time. Differences in the timing of investigations may be one explanation of inconsistent findings across studies. The low levels of problems reported in the current study may be explained by the two decades that separated the accident and the investigation.

In a survey study [25] estimating Chernobyl-related anxiety among Norwegians in the first two months following the accident, the anxiety and stress produced by the accident only reached clinical levels for about 1% of the respondents. Studies that have investigated the effects of toxicological disasters provide evidence of a significant increase in the number of legal abortions [8], but there was no rise in legal abortions in Norway in the year following the Chernobyl accident [26]. These findings suggest that even though the accident and its consequences in Norway were well known, Norwegians were less worried about the potential impact of exposure to Chernobyl fallout.

Johnson and Galea [10] have described risk factors associated with mental health problems after disasters. Among these are: direct exposure to the disaster; the degree of exposure to and direct threat from the disaster; participation in rescue and cleanup; media exposure; indirect consequences of the disaster (such as relocation or residential problems and community destruction); proximity to the disaster; being in the disaster-affected area at the time of the disaster; alcohol-related problems since the disaster; events since the disaster; negative life events; demographics; low-medium socioeconomic status or education level; social factors; limited post-disaster help; perceived similarity to victims; and perceived risk.

The risk factors mentioned above were higher for the exposed population from the former Soviet Union than for the Norwegian population living in exposed areas, with the most obvious difference being proximity to the disaster. The exposed population in the former Soviet Union experienced a lack of information, disorderly evacuation, conflicts over housing and benefits, and inadequate medical care [5]. Because of the collapse of the former Soviet Union, there were dramatic changes in the socioeconomic environment as well [23]. This can explain the fact that even though researchers did not document differences between evacuees and controls in Kiev, CBCL problem scores were generally high for both groups [5,7].

The Norwegian authorities provided systematic measures of ionizing radiation, adequate information about the potential dangers, restrictions on certain kinds of polluted foods, readily available health care and economic compensation for farmers in affected areas. There was no evacuation as a result of the accident. As in the rest of Norway, the participants in this study came from families that enjoyed a high living standard and social security. Higher social class, usually measured by education and income, is associated with better mental health outcomes after accidents [8]. It is likely that these factors have served as a protective buffer against the potentially harmful psychological effects of the accident on Norwegians. The perception of physical and psychological distance from the accident has probably had a protective effect as well.

### Limitations

There are limitations to the present study that need to be emphasized. First, the fact that there were no available accurate measures of radiation exposure to each individual. Second, this study investigated a small population within a limited age range, which meant that the sample size was small. It would have been useful to have a sample that represented all in utero exposed adolescents in the population and a suitable comparison group. Unfortunately, there is a lack of Norwegian data regarding the age group we are studying, and no national norms. On the basis of previous studies, one would expect to find low Norwegian problem scores.

Third, the number of nonresponders in the present study is high. Studies have shown that bias is likely to be introduced through nonresponse by the exclusion of participants who report higher levels of problems [17]. When we look at the demographic characteristics of the nonresponders, including a screening of psychological disorders (MINI SCID), no significant differences were found on these measures. Even though we may assume that the nonresponders would report slightly more problems, it is unlikely that they would be significantly different regarding emotional and behavioral problems. However, the lack of data in the current study makes generalization difficult.

Cultural differences in the levels of problems and in response style can make cross-cultural comparisons between studies difficult. A finding across cultures is higher problem scores in children from lower socioeconomic status (SES), particularly on Externalizing scores [27]. There are significant differences in SES between citizens from Norway and the former Soviet Union. Furthermore, the finding that adolescents usually report more problems than parents [28] seems to be particularly pronounced in Norway and Sweden, with very low scores on the CBCL and higher scores on the YSR [28].

Differences in SES and culture could potentially explain the discrepancy between the findings of the current and previous investigations in the former Soviet Union. However, because problem scores in previous studies were more highly rated by mothers in the prenatally exposed groups than mothers in the control groups, we do not think SES and cultural differences in response style fully explain the observed differences between the current and previous Chernobyl studies.

A major strength of this study was the access to demographic characteristics of the nonresponders, including a screening of psychological disorders (MINI SCID). In addition, participants included in this study were drawn from areas that enjoyed a high standard of living. In contrast to other studies, poverty did not affect the results.

## Conclusion

The results presented here demonstrate a contrast to previous studies of children exposed prenatally to Chernobyl radiation. In spite of previous reports of subtle cognitive deficits in prenatally exposed Norwegians, self-reported problems were few, and problems reported by the mothers even fewer. This is an unusual pattern, compared with other studies of prenatally exposed children from the former Soviet Union. Most studies have reported some kind of problems as measured by self report, and from the mothers of in utero exposed children. A possible explanation for this discrepancy between investigations is that the mothers of the Norwegian participants experienced less Chernobyl-related anxiety, due to fortunate circumstances in Norway and perceived physical and psychological distance from the disaster. This may have served as a buffer against a negative impact on the psychological health of both mother and child. Other explanations of the few problems reported may be the passage of time since the disaster, cultural differences between participants in the different investigations and/or lack of data in the current study. This study confirms previous findings of low levels of child behavior problems in Norway. The data do not suggest negative long-term effects on emotional and behavioral functioning as reported by these adolescents and their mothers in relation to in utero exposure to Chernobyl fallout.

## Competing interests

The authors declare that they have no competing interests.

## Authors' contributions

KSH contributed to the design and with acquisition of data, analysis and interpretation of data, and drafted and revised the manuscript. SM contributed with conception and design of the study and revised the paper for important intellectual content. KS contributed with analysis and interpretation of data and supervised drafts and revisions of the article. BRR contributed with conception and design of the study and supervised the analysis and interpretation of the data and drafts and revisions of the article. All authors read and approved the final manuscript.
